# Comparison of Acute Physiology and Chronic Health Evaluation (APACHE) II, Physiological and Operative Severity Score for the Enumeration of Mortality and Morbidity (POSSUM), and Surgical Apgar Score in Patients Undergoing Emergency Laparotomy

**DOI:** 10.7759/cureus.109365

**Published:** 2026-05-21

**Authors:** Sudhir Marahanumaiah, Preksha V Reddy, Kailash Jagannath, Nikita Galani, Nitish Suresh

**Affiliations:** 1 Department of General Surgery, Kempegowda Institute of Medical Sciences, Bangalore, IND; 2 Department of General Surgery, Kempegowda Institute of Medical Sciences Hospital, Bangalore, IND

**Keywords:** apache ii, emergency laparotomy, morbidity, mortality, possum, risk prediction, surgical apgar score

## Abstract

Background and aim: Surgical risk scoring is essential for predicting postoperative outcomes, guiding ICU admission, and planning interventions. In emergency laparotomies, appropriate scoring systems aid prognostication, risk‑adjusted audits, and quality improvement initiatives. This study aimed to compare the accuracy of the surgical Apgar score (SAS), Acute Physiology and Chronic Health Evaluation (APACHE) II, and Physiological and Operative Severity Score for the Enumeration of Mortality and Morbidity (POSSUM) in predicting morbidity and mortality among patients undergoing emergency laparotomy at Kempegowda Institute of Medical Sciences (KIMS) Hospital, Bangalore.

Methods: This prospective observational study included 65 adult patients undergoing emergency laparotomy between July 2023 and December 2024. Patients were assessed using parameters specific to SAS, APACHE II, and POSSUM. Postoperative complications were monitored on days three, seven, and 15, and graded using the modified Clavien-Dindo classification. Receiver operating characteristic (ROC) curve analysis was used to evaluate predictive validity.

Results: Morbidity occurred in 16 patients (24.6%) and mortality in 12 patients (18.5%). Higher SAS values were associated with fewer complications and lower mortality, whereas patients who died had notably lower scores. APACHE II demonstrated strong predictive accuracy, with higher scores significantly associated with adverse outcomes. POSSUM also effectively predicted morbidity and mortality, though with a tendency to overestimate risk in some cases.

Conclusion: All three scoring systems demonstrated predictive utility. APACHE II was the most reliable predictor of mortality, POSSUM showed greater accuracy for morbidity, and SAS, despite its simplicity, proved effective for both morbidity and mortality. Its ease of use supports SAS as a practical tool for guiding postoperative care decisions.

## Introduction

Emergency laparotomy refers to any unplanned open abdominal surgery, performed across all age groups and health states. Reported mortality averages 10-18%, markedly exceeding rates seen in elective procedures [1]. Thirty‑day mortality ranges from 6.2% in the United Kingdom’s National Emergency Laparotomy Audit (NELA) to 9.2% in the Australian and New Zealand Emergency Laparotomy Audit-Quality Improvement (ANZELA‑QI), reflecting differences in patient populations, pathology, surgical techniques, and outcomes [2,3]. Global access to emergency surgical care remains unequal, with low‑income countries bearing the highest burden of mortality [4]. This heterogeneity underscores the need for reliable risk prediction tools to guide operative decisions, support patient and family counseling, and optimize the use of scarce resources such as operating theatres and critical care beds [5]. Structured audits, including NELA, ANZELA‑QI, and the American College of Surgeons National Surgical Quality Improvement Program (ACS NSQIP), demonstrate an international consensus on using validated risk tools to improve survival and reduce complications. While India does not yet have a national audit, institutional initiatives at tertiary centers are emerging [4]. Risk scoring systems applied pre‑ and postoperatively allow clinicians to stratify patients and anticipate adverse outcomes. Risk scores are diagnostic tools, not interventions. Their effectiveness in reducing mortality depends on whether health systems embed them into structured pathways, for example, mandatory preoperative scoring linked to ICU admission criteria, surgical team allocation, or enhanced recovery protocols [5]. Acute Physiology and Chronic Health Evaluation II (APACHE II), Physiological and Operative Severity Score for the Enumeration of Mortality and Morbidity (POSSUM), and the surgical Apgar score are widely used to predict morbidity and mortality. APACHE II and POSSUM provide structured assessments of patient condition and surgical risk, helping identify those most vulnerable to complications. However, no single preoperative tool has demonstrated clear superiority in emergency laparotomy [6].

## Materials and methods

This single‑center prospective observational study was conducted at the Kempegowda Institute of Medical Sciences, Bangalore, from July 2023 to December 2024, following approval by the Institutional Ethics Committee. Adults (> 18 years) undergoing emergency laparotomy who provided written informed consent were included. Exclusion criteria were trauma, transplantation, vascular surgery, re‑laparotomy, immunosuppression, and loss to follow‑up. Convenience sampling was employed. Sample size was calculated using the reported specificity of the surgical Apgar score (SAS) (81.1% for a cut‑off < 6 in predicting three‑month mortality), yielding 59 subjects; after accounting for a 10% non‑response rate, 65 patients were recruited.

Data were collected intraoperatively and from anesthesia records using a structured questionnaire covering sociodemographic and clinical variables, complication severity (Clavien-Dindo classification), and scoring parameters for SAS, Acute Physiology and Chronic Health Evaluation II (APACHE II), and Physiological and Operative Severity Score for the Enumeration of Mortality and Morbidity (POSSUM) [7]. The SAS was derived from the lowest mean heart rate, estimated blood loss, and lowest mean arterial pressure, stratifying patients into high (0-4), moderate (5-6), and low (7-10) risk groups. Blood loss was estimated using the gross formula, incorporating pre‑ and postoperative hemoglobin values, body weight, and transfusion units.

Routine preoperative evaluation included complete blood counts, blood sugar, liver and renal function tests, serum electrolytes, arterial blood gas analysis, electrocardiogram, and chest X‑ray. Patients were followed postoperatively on days three, seven, and 15, with complications recorded and graded using the modified Clavien-Dindo classification. Outcomes such as renal failure, deep vein thrombosis, infections, anastomotic leak, cardiac arrest, sepsis, shock, and death were correlated with SAS, APACHE II, and POSSUM scores.

Data were entered in Microsoft Excel (Redmond, WA: Microsoft Corp.) and analyzed using SPSS version 22 (Armonk, NY: IBM Corp.). Continuous variables were summarized as mean±standard deviation, with independent t‑tests used for group comparisons. Receiver operating characteristic (ROC) curves were constructed for each scoring system against mortality, with sensitivity, specificity, and predictive values calculated. An area under the curve (AUC) > 0.8 was considered good discrimination. A p < 0.05 was considered statistically significant.

## Results

Among 65 patients, 16 (24.6%) developed postoperative morbidity, and 12 (18.5%) suffered mortality. Most patients were aged 51-60 years, with 36 (55.4%) males. Hollow viscus perforation was the leading indication for emergency laparotomy, followed by duodenal ulcer perforation, perforated appendix, and acute intestinal obstruction. Comorbidities were common, with type 2 diabetes mellitus being the most prevalent at 24 (36.9%), followed by hypertension and ischemic heart disease. Malignancy was rare, and most patients fell into the moderate severity category. Postoperative complications were documented on days three, seven, and 15. Superficial wound infection was most frequent, while wound disruption, anastomotic leak, and death became more prominent by day 15.

Patients with morbidity had significantly higher APACHE II and POSSUM scores and lower SAS scores compared to those without morbidity. Mortality was similarly associated with higher APACHE II and POSSUM scores and lower SAS scores. Mean APACHE II scores were 22.17 (n = 12) in deceased patients vs. 9.64 (n = 53) in survivors. POSSUM scores averaged 44.75 (n = 12) in deceased patients and 31.15 (n = 53) in survivors. SAS scores were significantly lower in patients who died (mean: 4.00, n = 12) compared to survivors (mean: 7.77, n = 53).

Scoring system performance

Surgical Apgar Score

The mean surgical Apgar score (SAS) was 6.56 in patients without morbidity, compared with 7.24 in those with morbidity. This difference was statistically significant on an independent sample t-test (t = -1.84, p = 0.0401). Patients who survived had a significantly higher mean SAS (7.77) compared to those who died (4.00). This difference was highly statistically significant (t = 8.24, p = 0.001). Despite poor discrimination, SAS demonstrated perfect sensitivity for morbidity, making it useful for rapid screening, though specificity was low (Table 1).

**Table 1:** Performance metrics of SAS for morbidity and mortality. SAS: surgical Apgar score; AUC: area under the curve

Variable SAS	AUC	p-Value	CI lower	CI upper	Optimal cutoff	Sensitivity	Specificity
Morbidity	0.264	0.0401	0.160	0.379	5.0	1.0	0.204
Mortality	0.050	0.001	0	0.162	5.0	0	1.0

APACHE II

Patients who developed postoperative morbidity had a higher mean APACHE II score (12.18) than those without morbidity (11.25), and this difference was statistically significant on an independent samples t-test (t = -0.80, p = 0.0396). With respect to mortality, patients who expired had markedly higher mean APACHE II scores (22.17) than survivors (9.64), and this difference was found to be highly statistically significant (t = -20.11, p = 0.001), indicating a strong association between higher APACHE II scores and adverse outcomes. Its comprehensive assessment supports accurate outcome prediction and reinforces its role in critical care (Table 2).

**Table 2:** Performance metrics of APACHE II for morbidity and mortality. APACHE II: Acute Physiology and Chronic Health Evaluation II; AUC: area under the curve

Variable APACHE II	AUC	p-Value	CI lower	CI upper	Optimal cutoff	Sensitivity	Specificity
Morbidity	0.538	0.0396	0.395	0.676	12.0	0.500	0.653
Mortality	1.0	0.001	1.0	1.0	20	1.0	1.0

POSSUM

The mean POSSUM score was significantly higher among patients who developed postoperative morbidity (34.24) compared to those without morbidity (31.88), with an independent sample t-test demonstrating statistical significance (t = -1.38, p = 0.0017). Similarly, patients who died during the postoperative period had significantly higher mean POSSUM scores (44.75) than survivors (31.15), and this association was highly statistically significant (t = -4.54, p = 0.001). While less predictive than APACHE II, POSSUM balanced sensitivity and specificity, positioning it as an intermediate tool between SAS and APACHE II (Table 3). Table 4 summarizes the postoperative complications observed on days three, seven, and 15.

**Table 3:** Performance metrics of POSSUM for morbidity and mortality. POSSUM: Physiological and Operative Severity Score for the Enumeration of Mortality and Morbidity; AUC: area under the curve

Variable POSSUM	AUC	p-Value	CI lower	CI upper	Optimal cutoff	Sensitivity	Specificity
Morbidity	0.48	0.0017	0.326	0.598	26.0	1.0	0.122
Mortality	0.48	0.001	0.704	0.992	38	0.833	0.868

**Table 4:** Postoperative complications observed on days three, seven, and 15.

Variables	Category	n	%
Day 3	Anastomotic leak	3	4.62
	Massive blood transfusion	1	1.54
	Sepsis and shock	3	4.62
	Superficial wound infection	7	10.77
	Death	3	4.62
	None	48	73.85
Day 7	Anastomotic leak	2	3.08
	Superficial wound infection	11	16.92
	Sepsis and shock	5	7.69
	Deep organ space infection	1	1.54
	Deep vein thrombosis	3	4.62
	Massive blood transfusion	1	1.54
	Wound disruption	3	4.62
	Death	2	3.08
	None	37	56.92
Day 15	Wound disruption	7	10.77
	Death	7	10.77
	None	51	78.46

**Figure 1:**
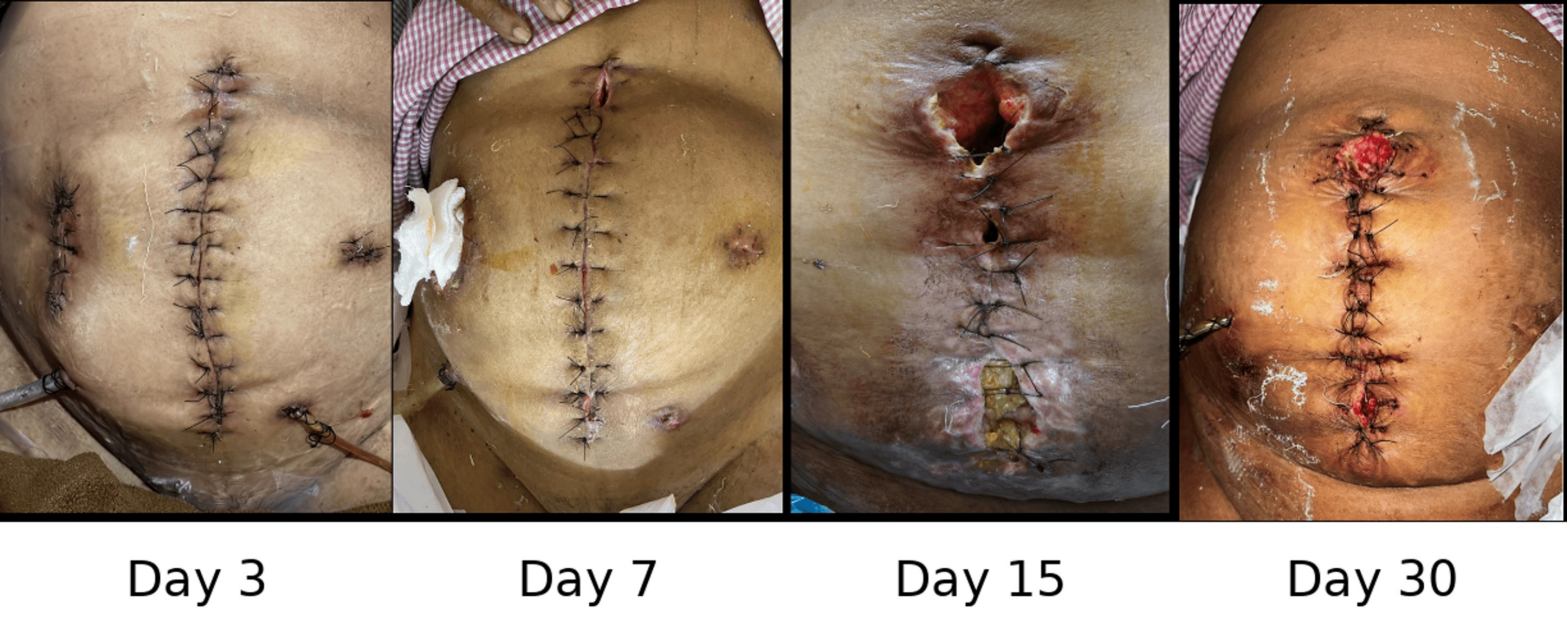
Seventy-four-year-old female with malignant stricture with large bowel obstruction. Clinical timeline of postoperative wound progression: day 3 - healthy wound with no erythema or discharge; day 7 - wound gaping; day 15 - wound dehiscence with fecal discharge; day 30 - wound showing signs of healing. The patient had a SAS of 4, APACHE II score of 24, and POSSUM score of 38. SAS: surgical Apgar score; APACHE: Acute Physiology and Chronic Health Evaluation; POSSUM: Physiological and Operative Severity Score for the Enumeration of Mortality and Morbidity

**Figure 2:**
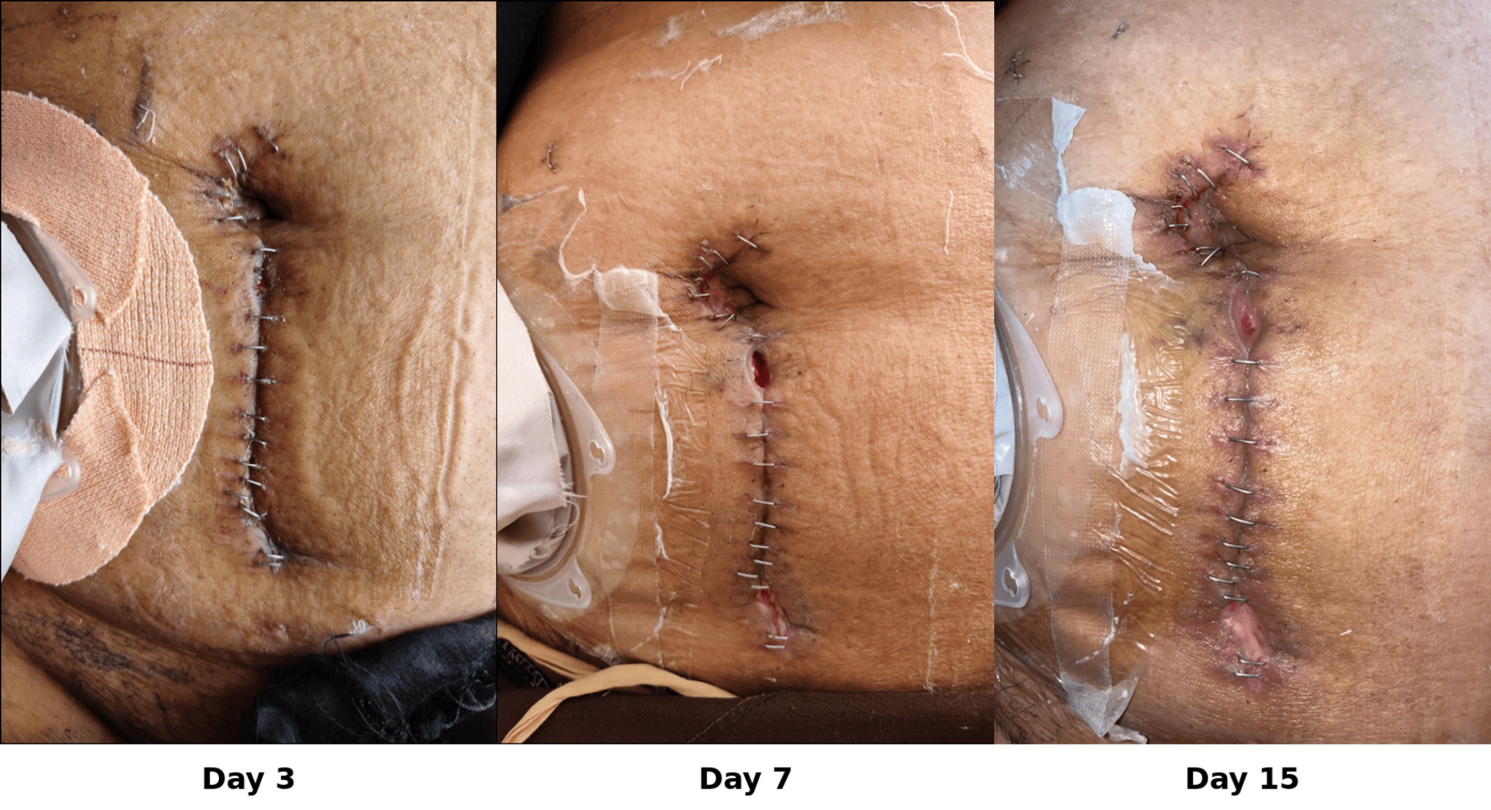
Fifty-year-old female with large bowel obstruction with malignant stricture. Clinical timeline of postoperative wound healing: day 3 - healthy wound with no erythema or discharge; day 7 - minor wound gaping; day 15 - healed wound. The patient had a SAS of 7, APACHE II score of 11, and POSSUM score of 32. SAS: surgical Apgar score; APACHE: Acute Physiology and Chronic Health Evaluation; POSSUM: Physiological and Operative Severity Score for the Enumeration of Mortality and Morbidity

Figure 1 shows postoperative wound pictures of a patient with a malignant stricture causing large bowel obstruction. Postoperative complications included sepsis, anastomotic leak, deep/organ space infection, shock, and death. Figure 2 shows postoperative wound pictures of a patient with large bowel obstruction due to a malignant stricture. Postoperative complications included superficial surgical site infection.

**Figure 3:**
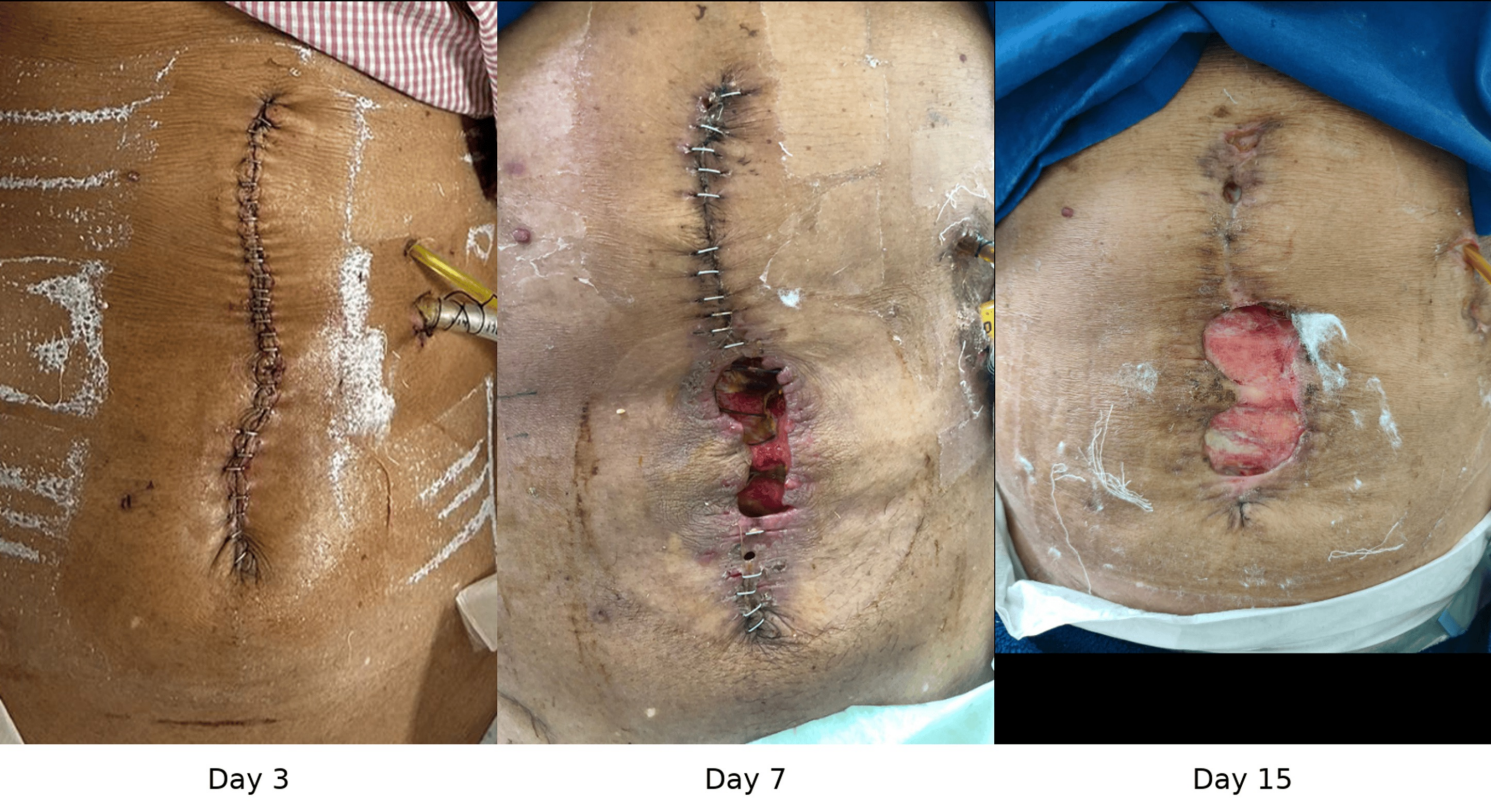
Forty-year-old male with hollow viscus perforation. Clinical timeline of postoperative wound healing: day 3 - healthy wound with no erythema or discharge; day 7 - partial wound dehiscence; day 15 - healed wound with scar formation. The patient had a SAS of 7, APACHE II score of 10, and POSSUM score of 28. SAS: surgical Apgar score; APACHE: Acute Physiology and Chronic Health Evaluation; POSSUM: Physiological and Operative Severity Score for the Enumeration of Mortality and Morbidity

Figure 3 shows postoperative wound pictures of a patient with hollow viscus perforation. Postoperative complications included sepsis and wound disruption. All three scoring systems demonstrate very large to extremely large effect sizes for mortality prediction, with APACHE II showing the strongest discriminatory power, followed by POSSUM and SAS (Table 5). All three scores show small effect sizes for morbidity prediction, indicating limited discriminative ability for postoperative morbidity in this cohort (Table 6).

**Table 5:** Cohen’s d effect size analysis demonstrating the predictive strength of SAS, APACHE II, and POSSUM scores for mortality in patients undergoing emergency laparotomy. SAS: surgical Apgar score; APACHE: Acute Physiology and Chronic Health Evaluation; POSSUM: Physiological and Operative Severity Score for the Enumeration of Mortality and Morbidity

Scoring system	Cohen’s d	Effect size interpretation
SAS	2.97	Very large
APACHE II	4.54	Extremely large
POSSUM	2.1	Very large

**Table 6:** Cohen’s d effect size analysis demonstrating the predictive strength of SAS, APACHE II, and POSSUM scores for morbidity in patients undergoing emergency laparotomy. SAS: surgical Apgar score; APACHE: Acute Physiology and Chronic Health Evaluation; POSSUM: Physiological and Operative Severity Score for the Enumeration of Mortality and Morbidity

Scoring system	Cohen’s d	Effect size interpretation
SAS	0.35	Small to moderate
APACHE II	0.16	Small
POSSUM	0.28	Small

## Discussion

The present study evaluated the predictive validity of APACHE II, POSSUM, and surgical Apgar score (SAS) in emergency laparotomy.

SAS

Patients with morbidity had higher SAS (7.24 vs. 6.56), while those who died had lower scores (4.0 vs. 7.77). Although SAS showed statistical significance, its predictive accuracy was poor (AUC = 0.264 for morbidity, 0.050 for mortality). Similar limitations were noted by Shaikh and Akther, who found that low SAS strongly predicted complications, and by Mane et al., who found an increased risk with SAS < 4 [8,9]. In contrast, Onen et al. reported fair discriminatory ability (AUC ~ 0.75), and Ngarambe et al. found SAS useful for mortality prediction (AUC = 0.79) [10,11]. Thus, SAS remains a rapid screening tool but should not be used standalone.

APACHE II

This score demonstrated robust predictive power, especially for mortality (AUC = 0.958). Deceased patients had significantly higher scores (22.17 vs. 9.64). Similar findings were reported by Sreeharsha et al. [12], where higher APACHE II correlated with mortality, and Nag et al., who showed an AUC of 0.965 with strong sensitivity and specificity [13]. Hansted et al. noted that the APACHE II score predicted mortality moderately and admission to the intensive care unit poorly in emergency abdominal surgical patients [14].

POSSUM

Scores were higher in morbid (34.24 vs. 31.88) and deceased patients (44.75 vs. 31.15), with moderate predictive accuracy (AUC = 0.48). Shekar et al. found POSSUM overestimated morbidity and mortality but remained statistically significant, while Kumar et al. reported no significant difference between observed and predicted outcomes [15,16]. Yadav et al. and González-Martínez et al. also highlighted its moderate accuracy [17,18]. POSSUM thus serves as an intermediate tool, balancing sensitivity and specificity, particularly for mortality prediction.

Systematic reviews have further validated these findings. A meta‑analysis by Tekkis et al. on gastrointestinal surgery outcomes confirmed that POSSUM and P‑POSSUM are reliable for morbidity prediction but tend to overestimate mortality, whereas APACHE II provides more accurate mortality estimates in emergency surgical patients [19]. Another systematic review of perioperative risk scores by Prytherch et al. emphasized that no single scoring system is universally superior and recommended a multi‑system approach combining APACHE II, POSSUM, and simpler bedside tools, such as SAS, for comprehensive risk stratification [20].

Overall, APACHE II emerged as the most reliable scoring system, while POSSUM provided moderate accuracy, and SAS offered simplicity but limited predictive value. Combining these tools may enhance risk stratification and guide aggressive management in high‑risk patients.

A key takeaway is the necessity of a multifaceted approach to risk assessment. Reliance on a single scoring system may not provide a complete picture; integrating elements from multiple tools could enhance decision‑making and improve patient outcomes. This study was limited by its single‑institution design and modest sample size, which may introduce demographic and institutional biases. Surgeon expertise, intraoperative complications, and long‑term follow‑up were not extensively analyzed. Larger multicenter studies are needed to validate these findings across diverse populations. Integration of machine learning algorithms with traditional scoring systems may further refine risk prediction and enhance clinical utility.

## Conclusions

Receiver operating characteristic (ROC) curve analysis showed that APACHE II had the highest predictive accuracy for mortality, followed by POSSUM with moderate accuracy. SAS had limited predictive power, but significantly showed high sensitivity for predicting morbidity. To summarize, in our study, APACHE II emerged as the most reliable scoring system for predicting postoperative outcomes in emergency laparotomy, with POSSUM scoring system showing moderate predictive power. Although SAS demonstrated low overall accuracy, its simple calculation, fewer parameters, and high sensitivity make it a useful preliminary screening tool in resource-constrained settings.
